# Improvement in Tuberculosis Outcomes With a Combined Medical and Social Approach

**DOI:** 10.3389/fmed.2019.00135

**Published:** 2019-06-21

**Authors:** Jesica Mazza-Stalder, Emilie Chevallier, Onya Opota, Ana Carreira, Katia Jaton, Eric Masserey, Jean Pierre Zellweger, Laurent Pierre Nicod

**Affiliations:** ^1^Pulmonary Division, Lausanne University Hospital, University of Lausanne, Lausanne, Switzerland; ^2^Vaud Lung Association, Lausanne, Switzerland; ^3^Institute of Microbiology, Lausanne University Hospital, University of Lausanne, Lausanne, Switzerland; ^4^Public Health Department, Lausanne, Switzerland; ^5^Swiss Lung Association, TB Competence Center, Bern, Switzerland

**Keywords:** tuberculosis outcomes, treatment adherence, tuberculosis in Switzerland, directly-observed therapy, tuberculosis social approach

## Abstract

**Setting:** Studies performed locally in Switzerland in the late eighties reported unsatisfactory treatment outcomes. Better outcomes were observed since the introduction of directly observed therapy (DOT) in the late nineties and improvement in social support in recent years.

**Design:** retrospective study of treatment outcomes for all tuberculosis (TB) patients notified in Vaud County (VD), Switzerland, between, 1st of January 2010 and 31st of December of 2014.

**Results:** 375 patients were notified in VD during the study period. The global outcome was successful in 90.1% of patients (338/375). In 183 culture and PCR positive pulmonary TB, the documented cure rate was 57.9% (106/183), and the treatment completion was 59/183 (32.2%), i.e., a treatment success of 90.2%. DOT was applied globally in 234/375 (62.4%) and in 64/67 of the asylum seekers (AS) (95.5%) followed at the dispensary. Treatment outcomes were successful in 60/67 (89.6%) AS.

**Discussion:** Improvements in tuberculosis outcomes resulted not only from the introduction of DOT in VD in the nineties but also from a change in the management, with increased attention to the social problems faced by the migrants.

**Conclusion:** A combined medical and social approach of TB care in VD improved treatment outcomes.

## Introduction

In Switzerland in 2017, 554 cases of TB were notified to the Swiss Federal Office of Public Health (SFOPH), resulting in a notification rate of 6.5 cases per 100,000 population (1); this rate is fairly stable. Of these TB patients ~23%, are from Swiss origin and around 77% are from foreign-origin, asylum seekers (AS) account for 34% of the foreign-born patients. Among others, the efficient management of TB, as detailed in the END TB strategy of the WHO, implies rapid diagnosis, high cure rate, and avoidance of failure, relapses, and interruptions before the scheduled end of treatment (2, 3). To guarantee that TB cases are rapidly diagnosed, access to health care must be universally offered, and avoid stigmatization (4). Adherence is essential for treatment completion, TB prevention and care, and thus one of the major determinants of treatment outcomes (5). In Switzerland, studies performed in the late eighties described unsatisfactory treatment outcomes with a success rate in VD County not exceeding 70% in patients with complete documentation (63% if patients without documentation are included) (6). A countrywide survey revealed better results but confirmed that the success rate among some subgroups of foreign-born patients, for instance undocumented migrants, was below the expected range (7). Local studies demonstrated that the provision of directly observed therapy (DOT) and a better training of the health care workers caring for TB patients had an important impact on the cure rate. Since then, VD County invested more resources in the management of TB. Our aim is to present the integrated medical and social approach implemented in VD County for the management of TB, and the current outcomes.

## Methods

### Description of the Cantonal Tuberculosis Health Care System

VD County has a population of 794,000 inhabitants with 33% of foreign-born residents and notifies around 65 cases of TB per year. TB patients are mostly followed by specialists at the TB clinic (TB dispensary or DAT) of the University Hospital of Lausanne. TB notification to the cantonal health authority is mandatory in Switzerland. The physician in charge of the patient declares the new TB cases to the Cantonal health officer who transfers the information to the SFOPH. Diagnostic laboratories, independently, also notify to the cantonal health authority all new strains of *M. tuberculosis* identified. The TB nurses from the Lung association (a Non Governmental Organisation caring for patients with chronic respiratory diseases) carry out contact investigations if ordered by the cantonal health officer. A medical advisor (a lung specialist) works at both the Lung association and the TB dispensary (DAT) and is available for questions regarding TB diagnosis, treatment, follow up, and contact tracing. Notification of the issue of treatment is requested by SFOPH since 2016 for all TB patients.

### Study Population, Data Collection, and Ethics Approval

All TB patients notified in VD County between, 1st of January 2010 and 31st of December of 2014 were eligible for inclusion in our study. Patient's data were obtained using the VD Lung Association records and the electronic medical records at the DAT. A common excel table was elaborated to analyze the data. End of treatment notification results forms for pulmonary culture positive patient were obtained, missing information was obtained directly from medical doctors in charge of the patients (mostly lung or infectious disease specialists) or from the final reports on the electronic medical records of the Hospital. We collected data on age, nationality, type of TB (pulmonary [PTB] or extra-pulmonary [EPTB]), new or retreatment cases, legal resident status, tuberculosis resistance pattern, and follow-up 2 years after the end of treatment. Authorization was obtained from the Ethics Committee of VD County *(CER-VD 2017-02300)* to review the clinical records and publish the analyzed data. Written informed consent was not required. All patient identifiable information was anonymized.

### Outcomes

Treatment outcomes were defined according to the WHO 2013 revised definitions (8). Successful outcomes included cure (documented with bacteriological conversion in culture positive pulmonary TB) and treatment completion (treatment completed without bacteriological confirmation). Unsatisfactory outcomes included treatment failure, lost to follow up and deaths (deaths due to TB or not due to TB). Potentially unsatisfactory outcomes included “transferred out” cases for whom the treatment outcome was unknown.

### Microbiology

Acid fast bacilli detection was achieved through a fluorescent auramine-thiazine red staining according to international guidelines (9). Detection of *M. tuberculosis* complex was achieved either using a real-time PCR targeting the IS6110 on the molecular diagnostic platform of our laboratory of microbiology (10) or using the Xpert MTB/RIF (11). Mycobacterial culture and drug susceptibility test for first-line drugs were achieved in Mycobacteria Growth Indicator Tube. Drug sensitivity testing for second-line anti-TB drugs for MDR- and XDR-TB was performed at the National Reference Center for Tuberculosis (Zurich, Switzerland).

### Treatment Administration

Whenever possible, and at least for all patients recently arrived in Switzerland and during the intensive phase, the treatment was administered under DOT, supervised by nurses, be it by a daily visit to the DAT or to a pharmacy close to the living place or in the social environment of the patient. For well-integrated patients with stable living place and no risk of unexpected moving out of the region, the treatment during the continuation phase may have been under self-administration or by collecting the treatment in the pharmacy once a week (with a pill organizer).

### Social Support

Every patient with TB diagnosed in the County received a visit from a social worker during the isolation in hospital or within the first 2 weeks of treatment if the patient was at home. Enablers to adherence such as psychosocial, social or financial support were deployed when appropriate as suggested by European standards of tuberculosis care (12–14). Housing closer to the hospital was whenever possible obtained thanks to the networking between the social services and the different structures that provide housing for migrants (15). Interventions to assist and motivate the patients such as providing extra food vouchers, clothes or assistance to travel, were offered, when necessary. Missed appointments were re-scheduled systematically to avoid interruptions while on treatment as proposed by others (16). In case of drug dependency, methadone programs were combined together with TB treatment and DOT (17). Patients missing one DOT appointment received a telephone call or a reminder letter in order to re-establish contact with the TB clinic. To exchange information on DOT, contact tracing, social and financial enablers to improve treatment adherence, we organized monthly meetings with all the different actors of TB care. Enablers and facilitators of treatment adherence are listed in Table 1.

**Table 1:** Social enablers applied at the DAT to facilitate adherence.

**Examples of enablers and incentives applied at the dispensary (DAT)**
**Improvements at the DAT**
Increasing the number of nursing staff Better training of the nurses (organized at a national level by the Swiss Lung Association) Hiring a Social Worker (since 2011) Close collaboration between the nurses of the Vaud Lung Association and the DAT Extended opening hours at the DAT (Monday to Friday 8 to 18 h, weekend open pharmacy and lung specialist on call) Missed appointments /Reschedule of appointments
**Improvements in the patient's care**
Increasing use of systematic DOT in migrants and vulnerable population Working on psychosocial aspects in close collaboration with psychiatrics and social assistants if needed or centers for alcohol or drug abuse/combined Methadone with anti-tuberculous drugs in DOT Facilitators of adherence (food vouchers, ticket transport or taxis when needed, closer housing, clothing when needed) Patient education (explaining the disease and the improvement while on treatment, tobacco prevention, and intervention for smoking cessation) Financial support (by the cantonal Public Health authorities) applied when necessary
**Improvements in communication**
Nurses speaking different languages/ Hiring translators Empathy, skills in community medicine, addressing TB related-stigma issues, discussing fears of deportation, and reassuring the patients Monthly team meetings regrouping nurses, lung and infectious disease specialists, cantonal medical officer, social assistant, health workers working at shelters for migrants Avoidance of deportation while on treatment (paper work with cantonal and federal migration and following the national law on epidemics)

## Results

Between January 2010 and December 2014, 375 patients were notified in VD, with a male predominance of 62.1%. The mean age was 38 years (range 1–98) for the whole group and 67 years (3–98) in Swiss patients. There were 288 foreign-born patients (77%) mainly Africans (26.4%), Western Europeans (16.2%), Eastern Europeans (15.7%), and 87 Swiss patients. TB was diagnosed for the first time in 269 patients (72%), 44 patients (12%) had been treated previously and no information on previous treatment was available for 62 patients (17%). Pulmonary tuberculosis (PTB) alone affected 222 patients (59.2%), extra-pulmonary-TB (EPTB) was diagnosed in 103 patients (27. 4%), and both PTB and EPTB was diagnosed in 50 patients (13.4%). Detailed patient's characteristics is reported in Table 2 and sites of disease (PTB and EPTB) in Table 3.

**Table 2:** Patient's demographics.

	***N***	**(%)**
**Gender**		
Male	233	(62)
Female	142	(38)
Total	375	(100)
**Origin**		
Switzerland	87	(23.2)
Africa	99	(26.4)
Europe	61	(16.2)
Eastern Europe	59	(15.7)
Eastern Mediterranean	20	(5.3)
Americas	18	(4.8)
Southeast Asia	15	(4)
Western Pacific	10	(2.7)
Southwest Asia	5	(1.4)
Central Asia	1	(0.3)
Total	375	100

**Table 3:** Sites of disease.

**Site of disease**	***N***	**(%)**
Pulmonary (PTB)	222	(59.2)
Extra-pulmonary (EPTB)	103	(27.4)
Both PTB+EPTB	50	(13.4)
**Total**	375	(100)
**EPTB sites**		
Extra thoracic lymph nodes	38	(24.8)
Pleural	37	(24.2)
Mediastinal lymph nodes	23	(15)
Disseminated	17	(11.2)
Bone and Spinal TB	16	(10.5)
Abdominal TB	6	(3.9)
Genitourinary tract	4	(2.6)
Cutaneous	4	(2.6)
SNC	4	(2.6)
Uveitis	2	(1.3)
Pharyngeal	2	(1.3)
**Total**	153	(100)

*P, Pulmonary; EP, Extra pulmonary; TB, Tuberculosis*.

### Microbiological Findings

Cultures were performed in 362 patients (96.5%), not done in 11 patients (3%), and information was missing for 2 patients (0.5%). Among all patients (PTB+ EPTB) culture was positive in 268/375 (71.5%). In patients with PTB only the culture and/or PCR was positive in 183/222 (82.4%) and for EPTB only patients we obtained 80/103 (77.6%) positive samples. We excluded from the analysis the culture positive among the group of PTB+EPTB (*n* = 41) because we couldn't differentiate the site from which the culture was positive. Among all the TB strains, 265 were *M. tuberculosis* (98.9%) and 3 were *M. bovis* (1.1%). Complete microbiological data is reported in Table 4.

**Table 4:** Microbiological findings.

**Microbiology**	***N*/total**	**(%)**
**Culture**		
Positive (all included)	268/375	(71.5)
Negative (all included)	94/375	(25)
Not done	11/375	(2.9)
Unknown	2/375	(0.6)
**Culture and/or PCR**		
Positive (all included)	281/375	(74.9)
Positive PTB	183/222	(82.4)
Positive PTB + EPTB	41/50	(82)
Positive EPTB (only)	80/103	(77.7)
Negative PTB	94/375	(25.1)
Negative EPTB	23/103	(22.3)
Negative PTB+EPTB	9/50	(18)

*TB, Tuberculosis; P, Pulmonary; EP, Extra-pulmonary*.

### Treatment Outcomes

**Figure 1:**
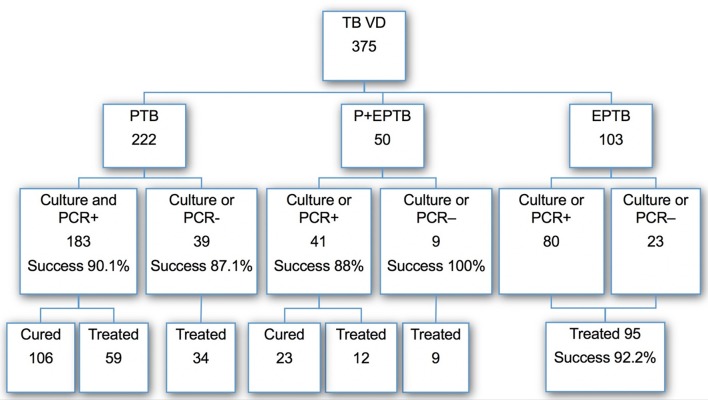
Distribution of TB cases, bacteriological results, and outcome of treatment in each category.

Globally, treatment was successfully completed in 338 patients (90.1%). Among 183 patients with culture or PCR positive PTB (excluding PTB+EPTB), including 10 MDR-TB, the documented cure rate was 106 (57.9%) and the treatment completion was 59 (32.2%), i.e., a global treatment success of 90.2% (*n* = 165). Among 50 patients with PTB+EPTB tuberculosis, the outcome was successful in 44 (88%). Among 103 patients with EPTB, the treatment completion was 95 (92.2%) (Figure 1).

Overall (PTB+EPTB) treatment success among all AS was 79/89 (87.6%). Most AS in VD County were treated at the DAT 67/89 (75.2%) 95.5% of them on DOT (64/67) with a treatment success of 60/67 (89.6%). DOT was applied in 234/375 (62.4%) of our entire study population. We observed no treatment failure. However, 19 patients had unsatisfactory outcomes (5.1%) and 18 potentially unsatisfactory outcomes (4.8%). Among these, we counted 7 persons lost to follow up (1.8%), 12 deaths (3.2%), and 18 transfers while on treatment (4.8%) (Table 5). Within the lost to follow up we counted 7 patients that were part of a very mobile population including 5 AS (2 Georgians, 3 Africans) and 2 foreign-born Europeans (Romanians). Among the 12 that died, 6 died from TB, and 6 died due to comorbidities. Among them 10 patients were Swiss (mean age 69 (49–89), the other two were foreign-born (mean age 52 (23–80). The 23-year-old patient that died while on treatment was an XDR-TB patient who died for causes other than TB. In two elderly Swiss patients (mean age 85) TB was not suspected before death and was diagnosed and notified only after an autopsy. Transmission of TB to health care workers occurred from these 2 undiagnosed patients, one to a nurse and one to a pathologist. Treatment outcomes in DOT vs non DOT is shown in Table 6. Outcomes by categories are shown in Table 7.

**Table 5:** Treatment outcomes.

**Outcomes**	**N/total**	**(%)**
**Successful outcomes**	338/375	(90.1)
Successful (overall PTB+EPTB)	338/375	(90.1)
Successful outcomes in culture or PCR positive PTB	165/183	(92.2)
Cured only PTB culture and/or PCR positive	106/183	(59.9)
Completed without bacteriological confirmation (PTB)	59/183	(32.2)
Successful outcomes in EPTB (completed)	95/103	(90.2)
Successful outcomes in AS (all included)	79/89	(88.8)
Successful outcomes in AS follow at DAT	60/67	(89.6)
Successful outcomes (PTB+EPTB) in Swiss patients	76/87	(87.3)
**Unsuccessful Outcomes**	19/375	(5.1)
Lost to follow-up	7/375	(1.8)
Failure	0/375	(0)
Deaths	12/375	(3.2)
**Causes of death**		
Deaths due to TB	12/6	(50)
Deaths due to causes other than TB (comorbidities)	12/6	(50)
Deaths in Swiss patients	12/10	(83.3)
Death in foreign patients	10/2	(16.7)
**List of comorbidities**		
Chronic Obstructive Pulmonary Disease	6/1	(16.6)
Heart failure	6/1	(16.6)
Immunosuppression/HIV	6/1	(16.6)
Immunosuppression /Anti-TNF therapy	6/1	(16.6)
Malignancy	6/2	(33.6)
**Potentially unsuccessful outcomes**	18/375	(4.8)
Transfer-out without knowledge of outcome	18/375	(4.8)

*Tt, Treatment; TB, tuberculosis; P, pulmonary; EP, extra-pulmonary; AS, Asylum seekers*.

**Table 6:** Outcomes DOT vs. no DOT.

**Outcome**	**DOT (*n* = 234)**	**No DOT (*n* = 141)**	**Total DOT + no DOT (*n* = 375)**
Cured	117	25	142
Treatment completed	104	92	196
Successful	221 (94.4%)	117 (82.9%)	338 (90.1%)
Transfer	8	10	18
Death	2	10	12
Lost to follow-up	3	4	7

**Table 7:** Outcomes by categories.

**Outcome**	**Female gender**	**Male gender**	**Mean age**	**PTB**	**P+EPTB**	**EPTB**	**Both (P+EP)**	**Swiss origin**	**Foreign -born**	**Asylum seekers**
Treatment success	134 (94%)	204 (87.5%)	42.0	199 (89.6%)	44 (88%)	95 (97%)	338 (90.1%)	76 (87.3%)	262 (90.9%)	79/89 (89%)
Unsuccessful treatment	8 (6%)	29 (12.5%)	49.8	23 (10.4%)	6 (12%)	8 (3%)	37 (9.9%)	11 (12.7%)	26 (9.1%)	10/89 (11%)
Total	142	233	–	222	50	103	375	87	288	89

### Follow-Up Visits After Treatment

We followed 178 patients for up to 2 years, 84.2% of these patients were followed at the DAT or at the university hospital in other units (pediatrics and infectious diseases). Relapses were observed in 2 patients, both during the first year after the end of treatment. No relapse was observed after the first year.

## Discussion

This retrospective study assessed TB treatment outcomes among patients in VD County during the period 2010–2014, and showed a global success rate of 90.1%. In culture positive pulmonary patients, the cure rate was similar (90.2%). Our success rate is comparable to studies performed at our institution after the implementation of DOT in the late nineties (88.9%) and with publications from St. Gallen in eastern Switzerland (87.5%) (18, 19). Our results are much superior to the historical results of 70% of treatment outcomes previously published by our own institution by Coulon et al. (6). In this study, and other local studies (20) treatment interruptions were more common among migrants and vulnerable populations because of change in place of living, deportation out of the country or disappearance in illegality for migrants not granted refugee status, and because of a high rate of non-adherence despite good access to health care in Switzerland. Adherence is one of the major determinants of treatment success (5). Among the measures considered for improving the adherence to TB therapy and the cure rate, the DOT is considered as one of the most crucial, since the seminal publication by Weis demonstrating that DOT contributed to the decrease in relapse and acquisition of drug resistance (21). Further studies supported the role of DOT as a method for improving the success rate of TB treatment (22). Recently, several other studies and analysis have questioned this dogma and demonstrated that DOT may not be the only element of success in TB treatment, but that the whole organization of patient care is contributing to the success (23, 24). The experience in our institution seems to confirm this, as the improvement in the cure rate resulted not only from the introduction of DOT in Lausanne in the nineties but also from a change in the management, with better training of specialized nurses, improvements in communication by hiring translators, re-organization of the appointment schedules, and above all increasing the attention to the social problems faced by the migrants and vulnerable populations (19). Indeed, since the nineties, a reinforcement of the existing personnel at the TB clinic was assured in VD County with extended opening hours as recommended in treatment guidelines (14, 25) and the social aspects were better considered with the inclusion of a social worker in the staff. Since most of the treatment interruptions in the late eighties occurred among AS followed outside the TB clinic by general practitioners lacking the necessary structure to take care of difficult social situations, in later years, we tried to follow this particularly vulnerable population at the DAT. We found that 75.2% of the AS were followed at the DAT and a DOT strategy was applied in 95.5% of them. Among the entire study population DOT was applied in 62.4%. As other programs showed before, we found that regulatory interventions (search for missing patient by police order) were rarely needed and were only applied in case of disappearance of infectious patients (26). Furthermore, thanks to an agreement between the SFOPH and the State Secretariat for Migration, migrants with TB, even those who are not granted asylum status, are no longer deported from the country during the treatment against their will, thus avoiding disappearance in illegality and treatment interruptions.

Like other groups we observed diagnostics delay among elderly people. Most of the deaths were among Swiss elderly patients. Atypical forms of Tb presentation among older patients and lack of suspicion of TB by doctors in elderly patients are well known (27). Diagnostic TB delays has been confirmed by a recent study conducted in Switzerland (28). In our study 2 very old Swiss patients (mean age 85) living in nursing homes were only diagnosed after death (autopsy). This finding underlines, as described by others, a real underappreciated problem with undiagnosed TB cases among older people with potential transmission in the community and particularly to health care workers (29).

Relapses were rare in our study group with only 2 patients in the year following the end of treatment.

This retrospective study has limitations, as we could not analyze prospectively if the improvements of outcomes and adherence were due to the health care and social interventions deployed among the most vulnerable populations. Another limitation of this study is that we described an observational cohort from a single center in comparison with a historical cohort only but without a control group, and that the role of each different components that helped improving the tuberculosis care (DOT vs. social support) could not be assessed properly in a prospective way. The strengths are the relatively high number of patients included in the analysis, the quality control of the daily work in the center and the good treatment outcomes obtained.

In summary, this study shows an improvement in treatment outcomes in VD County with a global success rate of 90.1% overpassing the expectations of 85% requested by the WHO. The patient-centered approach of TB care in VD County provides social protection with a high-quality standard of TB care and a scale-up compared to previous published outcomes.

## Ethics Statement

Commission cantonale d'Ethique sur la Recherche sur l'ê tre humain. CER-VD Project Number 2017-02300.

## Author Contributions

JM-S: design of the study, data collection and analysis, manuscript redaction. EC: data collection, manuscript revision. OO and JZ: data analysis and manuscript revision. KJ, EM, and LN: manuscript revision. All authors listed have made a substantial, direct and intellectual contribution to the work, and approved it for publication.

### Conflict of Interest Statement

The authors declare that the research was conducted in the absence of any commercial or financial relationships that could be construed as a potential conflict of interest.
